# Long-term survival of a patient with scalp angiosarcoma and multiple metastases treated using combination therapy: A case report

**DOI:** 10.3892/ol.2015.2919

**Published:** 2015-01-29

**Authors:** JUN YE, XIAO-FEN LI, YONG-DONG WANG, YING YUAN

**Affiliations:** 1 Department of Dermatology, Sir Run Run Shaw Hospital, Zhejiang University School of Medicine, Hangzhou, Zhejiang, P.R. China; 2 Department of Medical Oncology, The Second Affiliated Hospital, Zhejiang University School of Medicine, Hangzhou, Zhejiang, P.R. China; 3 Key Laboratory of Cancer Prevention and Intervention of Ministry of Education, The Second Affiliated Hospital, Zhejiang University School of Medicine, Hangzhou, Zhejiang, P.R. China

**Keywords:** angiosarcoma, scalp, comprehensive treatment

## Abstract

Angiosarcoma is a rare and deadly malignancy originating from the vascular endothelial cells. Surgery is the most effective method to cure this disease, but for metastatic angiosarcoma, a chemotherapy-based treatment is the main therapeutic choice. However, there is currently no standard chemotherapy regimen. The current study reports the case of a 66-year-old male with post-operative scalp angiosarcoma recurrence and multiple metastases. The patient obtained a complete response to first-line combination chemotherapy consisting of cyclophosphamide, epirubicin, vincristine and dacarbazine, with a progression-free survival time of eight months. After benefitting from subsequent comprehensive treatment including, cyclophosphamide, epirubicin, vincristine, dacarbazine, docetaxel, cisplatin, gemcitabine and radiotherapy and anti-angiogenic therapy, the patient obtained an overall survival time of 38 months following initial diagnosis.

## Introduction

Angiosarcomas are rare malignant sarcomas derived from vascular endothelial cells, and accounting for 1–2% of all soft-tissue sarcomas (1–5). In total, ~60% of angiosarcomas are cutaneous and the majority of these occur in the head and neck (1,3). Due to its high aggressiveness and multifocality, the prognosis of angiosarcoma is poor, with a reported five-year survival rate of ~35% in non-metastatic angiosarcoma cases (1,4,6). The majority of cases of recurrence (75%) occur within 24 months of local treatment (1). For local disease, radical resection and adjuvant radiotherapy are recommended (5), while for metastatic angiosarcoma, chemotherapy is the primary treatment choice, although there is no standard regimen (5).

The present study describes a case of scalp angiosarcoma, which was treated with surgery, followed by recurrence and multiple metastases, which were treated with post-operative combination chemotherapy and radiotherapy. The patient obtained an overall survival time of 38 months following initial diagnosis.

## Case report

A 66-year-old male was admitted to the Department of Dermatology of the Sir Run Run Shaw Hospital (Zhejiang University School of Medicine, Hangzhou, Zhejiang, China) in October 2007 with a four-month history of scalp masses. Upon physical examination, two masses without ulcers or tenderness were noted in the right temporoparietal area, measuring 3.0×3.0 cm and 1.5×1.5 cm, respectively. No superficial lymph nodes were palpable, and the rest of the physical exam was unremarkable. Brain magnetic resonance imaging (MRI) showed a subcutaneous soft-tissue mass with irregular margins in the right temporoparietal area, which was moderately enhanced upon enhanced scanning. Serum tumor marker levels were normal. Chest and abdominal computed tomography (CT) scans did not indicate distant metastases.

**Figure 1:**
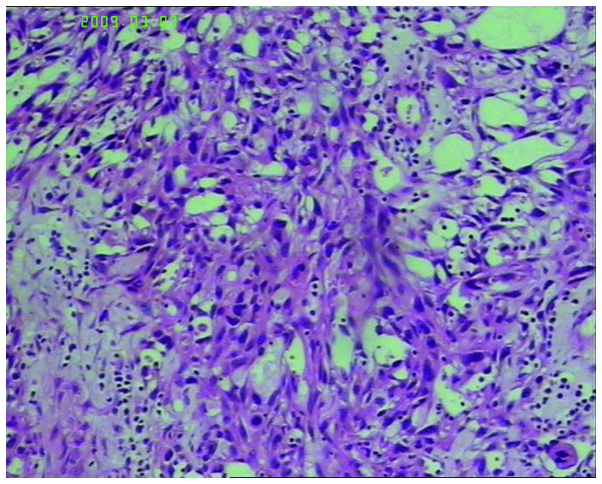
Sections stained with hematoxylin and eosin showing angiosarcoma of the scalp (original magnification, ×40).

Surgical excision was performed on October 31, 2007, following a pre-operative evaluation in the Department of Neurosurgery. The resection range was as large as 8.0×10.0 cm, so skin grafting with a free flap taken from the outer side of the right thigh was performed. The tumor did not invade the galea aponeurosis, and complete resection was achieved. The post-operative pathology revealed an infiltrative, irregularly configured vascular channel tumor. The tumor formed a pattern of vascular channels which were interlacing and anastamosing, which were lined with hyperchromatic endothelial cells, which exhibited mitotic activity. The pathological diagnosis was angiosarcoma of the scalp, with negative peripheral margins (Fig. 1). Adjuvant radiotherapy of the right parietal area was started at two months post-surgery, with a β-line dosage of 5,800 cGy/29 fractions for six weeks. Adjuvant chemotherapy was refused by the patient for personal reasons.

**Figure 2:**
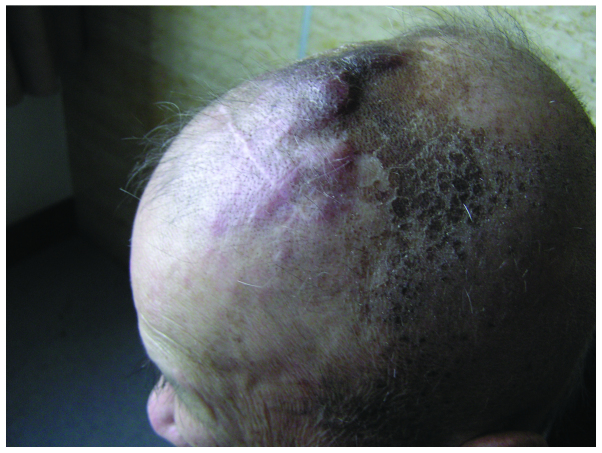
Recurrence of the scalp angiosarcoma.

In June 2008, more than seven months after the surgery, the patient was readmitted to the Department of Neurosurgery presenting with multiple scalp masses (Fig. 2). This was diagnosed as a post-operative recurrence of the scalp angiosarcoma. No evident distant metastasis was documented by chest and abdominal CT scans. The patient underwent an extended resection of the frontal and occipital scalp masses, measuring 6.0×2.5 cm and 1.0×1.0 cm, respectively, and a bilateral neck nodal dissection. The post-operative histopathological examination showed two scalp angiosarcomas with negative resection margins, and two out of eight lymph nodes were metastatic. Again, the patient refused post-operative chemotherapy.

Three months later in September 2008, a mass measuring 3×4 cm was found close to the left pulmonary hilum in a routine chest X-ray. A chest CT scan revealed a lobulated hilar mass of 2.5×2.5 cm in size in the left upper lobe, with enlargement of the mediastinal lymph nodes. A subsequent CT-guided core needle biopsy of the lung mass indicated metastatic angiosarcoma. The patient experienced no discomfort, such as coughing, dyspnea or chest distress. Following a diagnosis of post-operative lung metastasis of the scalp angiosarcoma, the patient received six cycles of first-line palliative chemotherapy consisting of 750 mg/m^2^ cyclophosphamide on day 1, 60 mg/m^2^ epirubicin on day 1, 1.4 mg/m^2^ vincristine on day 1 and 250 mg/m^2^ dacarbazine from day 1–5, repeated every three weeks. Chest CT showed disappearance of the lung mass, which indicated a complete response (CR) according to to the Response Evaluation Criteria in Solid Tumors (7). The progression-free survival (PFS) time following first-line chemotherapy was eight months, and the patient tolerated the chemotherapy extremely well. Grade 2 nausea and leucopenia were observed according to Common Terminology Criteria for Adverse Events (8). No severe adverse reactions, such as febrile neutropenia, or cardiac and renal dysfunction, were documented during the treatment process.

In April 2009, four months after the end of first-line chemotherapy, the patient presented with waist ache and numbness of the lower limbs. Brain MRI revealed a mass in the left parietal lobe. Whole-body bone single-photon emission CT showed abnormally enhanced metabolism in the vertebral joint of the 8th left rib, the 8th thoracic vertebra and the 1st lumbar vertebra. Further spinal MRI indicated signal changes in these bones, which were considered metastases. Chest CT showed no lesions. Consequently, a clinical diagnosis of post-operative recurrence of scalp angiosarcoma, with lung, brain and bone metastases, was established. The patient was referred to a radiation oncologist to receive brain, thoracic and lumbar vertebrae radiotherapy, with an X-ray dosage of 6 MV, 4,000 cGy/20 fractions for four weeks. The symptoms of waist ache and numbness of the lower limbs were greatly relieved upon the conclusion of radiotherapy. However, during the radiotherapy, a scalp nodule of 2 cm in diameter was found at the edge of the previous surgery area and a mass of 1.0×1.0 cm in size was found in the left parotid gland area, both without tenderness. The disease was considered as having progressed, so second-line chemotherapy was administered with a regimen of 75 mg/m^2^ docetaxel and 75 mg/m^2^ cisplatin on day 1, repeated every three weeks. Severe myelosuppression and liver dysfunction occurred following the first cycle of treatment. Therefore, the regimen was changed to 60 mg/m^2^ docetaxel on day 1 plus 30 mg/m^2^ cisplatin from day 1–2. The patient tolerated this well and received another five cycles of chemotherapy. In addition, twice daily administration of 100 mg oral thalidomide was prescribed throughout the treatment process and chemotherapy intervals. The scalp nodule and left parotid mass significantly reduced in size following the chemotherapy, and was considered to be a partial response (PR). The patient again obtained a PFS time of eight months for the second-line chemotherapy.

In March 2010, half a year after stopping the chemotherapy, an abdominal CT scan revealed multiple liver metastases during a routine follow-up. Chemotherapy was administered again with a regimen of 1.0 g/m^2^ gemcitabine on days 1 and 8, 75 mg/m^2^ cisplatin on day 1 and 15 mg endostatin on days 1–14, repeated every three weeks. Following six cycles of chemotherapy, the liver metastases were greatly reduced in size, which was again considered to be a PR. The patient continued to use endostatin as maintenance therapy. A PFS time of nine months was obtained for this third-line chemotherapy.

In December 2010, the disease progressed again. Best supportive care was provided, and the patient finally succumbed in February 2011, with an overall survival time of 38 months following initial diagnosis. Written informed consent was obtained from the patient’s family for publication of this case study and the accompanying images.

## Discussion

Angiosarcomas can occur in any region of the human body, and are subdivided into groups, including lymphoedema-associated angiosarcomas, cutaneous angiosarcomas, radiation-induced angiosarcomas, soft-tissue angiosarcomas and primary-breast angiosarcomas (9). The most commonly affected area is the face and scalp region, accounting for >50% of cutaneous angiosarcomas (3). As a rare and deadly malignant tumor, angiosarcoma of the head and neck accounts for <0.1% of all head and neck malignancies (10,11). Livingston and Klemperer first reported this disease in 1926 (12), and 38 years later, Jones described a series of nine cases occurring on the face and scalp (13). The prevalence of angiosarcomas is highest in elderly patients, however, they can develop at any age, and their distribution is similar between the two genders (5).

Due to the rarity and masquerading manifestations, angiosarcoma is often diagnosed late. In the early stage, cutaneous angiosarcoma can present as a bruise, or a typically multifocal raised purple-red papule, which may be mistaken for a benign lesion (9). With increasing tumor size, tissue infiltration, edema, tumor fungation, ulceration and hemorrhage can occur (9). If untreated, the tumor can reach ≥20 cm in size. Local invasion into the underlying calvarium and brain occurs frequently (14). Moreover, distant metastases often occur via the hematogenous or lymphatic routes, with the lung being the most commonly affected site (5,14). Other common sites include the liver, bones, soft-tissue structures and lymph nodes (1,4,15–18). The patient in the current study initially presented with a scalp mass. In the follow-up visits, distant metastases in the lung, brain and bones were sequentially detected.

The diagnosis of angiosarcoma mainly depends on the pathology. Under the microscope, pleomorphic and malignant endothelial cells are apparent in typical angiosarcoma tissues. In areas that are well differentiated, abnormal endothelial cells form functioning vascular sinusoids continuous with normal vascular channels. However, in areas of poor differentiation, the malignant endothelial cells form continuous sheets, usually with necrosis and hemorrhage (9,19,20). As it is difficult to diagnose angiosarcoma by its morphology, immunohistochemistry plays an important role in confirming the diagnosis. Typically, endothelial markers, including CD34, CD31, von Willebrand factor, Ulex europaeus agglutinin 1 and vascular endothelial growth factor are expressed (5). Von Willebrand factor, Ulex europaeus agglutinin 1 and CD31 are the most useful markers in poorly-differentiated cases (21).

Treatment modalities for angiosarcoma include resection with a wide margin, radiotherapy, immunotherapy and chemotherapy. Combination treatment is recommended to obtain an improved prognosis (22). For local disease, radical surgery in the form of a complete resection and adjuvant radiotherapy are suggested. However, clear margins are rarely obtained for scalp angiosarcoma (~20% cases) despite large resection areas (1,17). For this reason, post-operative radiotherapy is recommended. For metastatic angiosarcomas, however, chemotherapy is the primary treatment choice (5), although there have been no large randomized trials to confirm a standard chemotherapy regimen. In soft-tissue sarcomas, the main chemotherapy drugs are anthracyclines, ifosfamide and taxanes (5). Van Glabbeke *et al* performed a large meta-analysis of the effect of anthracycline-based chemotherapy on patients with soft-tissue sarcomas, and found an overall response rate of 26% and a median overall survival time of 51 weeks (23). Certain studies have reported similar response rates and worse survival times in angiosarcoma patients (4,24). Liposomal doxorubicin and paclitaxel have also been reported to be useful in angiosarcoma in a number of retrospective studies (25–28).

As aforementioned, angiosarcoma is derived from vascular endothelial cells. Based on the pathogenesis of angiosarcoma, certain antiangiogenic drugs, such as thalidomide (29–31), bevacizumab (32,33) and sorafenib (34), have been used and have shown promising therapeutic effects in angiosarcoma patients. The biological activities of thalidomide include antiangiogenesis, tumor necrosis factor-α (TNF-α) inhibition and immune stimulation. It is has previously been reported to be useful in angiosarcoma of the breast, small intestine and scalp (29–31). In the present case, chemotherapy combined with thalidomide and endostatin (Endostar) was used to control the disease. Endostar (YH-16), is a novel recombinant human endostatin that is expressed and purified in *Escherichia coli*, and acts specifically on neovascular endothelial cells to induce cell apoptosis, thereby playing an antiangiogenic role in treating cancer (35).

As the patient in the present study developed lung metastasis after surgery, first-line systemic chemotherapy consisting of cyclophosphamide, epirubicin, vincristine and dacarbazine was administered. The patient tolerated treatment well and achieved a CR after six cycles, proving that the combination regimen was effective for angiosarcoma. The PFS time for this first-line treatment was approximately eight months. Docetaxel plus cisplatin, combined with oral thalidomide were administered as second-line chemotherapy, and a PR was achieved with a PFS time of approximately eight months. Gemcitabine plus cisplatin combined with endostatin were administered as the third-line treatment, and a PR was achieved again with a PFS time of approximately nine months. The overall survival time of the patient was 38 months following initial diagnosis.

Angiosarcoma is a rare malignant sarcoma that is prone to local recurrence and distal metastasis. Radical surgery is the most useful method for treating this disease. Combination treatment based on chemotherapy is strongly recommended for advanced angiosarcoma, however, there is no standard chemotherapy regimen at present. The present study indicates that the three regimens of cyclophosphamide, epirubicin, vincristine and dacarbazine, docetaxel, cisplatin and thalidomide, and gemcitabine, cisplatin and endostatin are effective for treating angiosarcoma. Angiogenesis agents such as thalidomide and endostatin may be of potential use. Nevertheless, further large-scale prospective studies are required to improve the treatment of angiosarcoma.

## Abbreviations

MRI: magnetic resonance imaging
CT: computed tomography
CR: complete response
PFS: progression-free survival
PR: partial response
